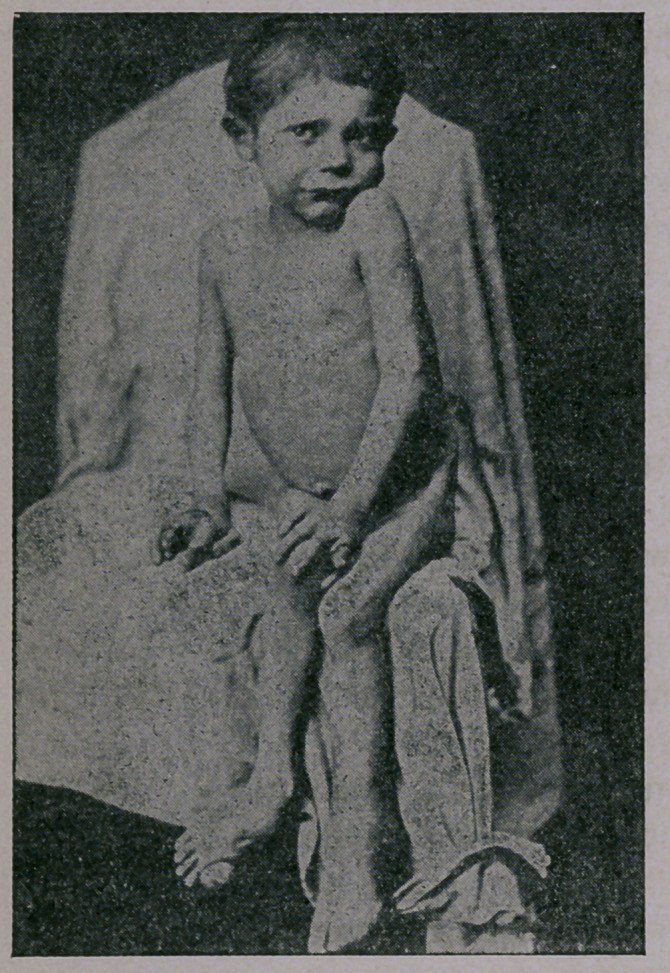# Vaccination in the Light of the Royal British Commission

**Published:** 1898-01

**Authors:** M. R. Leverson

**Affiliations:** Fort Hamilton, L. I., N. Y.


### VACCINATION IN THE LIGHT OF THE ROYAL BRITISH COMMISSION.

M. R. Leverson, M. D., Fort Hamilton, L. I., N. Y.

(Continued from November No., p. 441.)

Dr. Robert Cory was the next witness examined by the Commission. His examination was commenced on the 27th of November, 1889, was completed on the 4th of December, and he was recalled to enable him to explain portions of his former testimony on the 7th of May, 1890.

Q. 4,267–9. " Dr. Cory is Director of the animal and vaccine station, and was the first appointed on that institution. He is also one of the instructors in vaccination for the Local Government Board."

Q. 4,272. " Five calves are vaccinated every week."

Q, 4,273. " The lymph is generally taken from the calves five days after the vaccination. Human lymph is fully matured on the eighth day; but in the calf the course is more rapid." *

---

* This has an important bearing upon the pathological aspect of vaccination—probably it has some connection of cause and effect upon the increased prevalence of cancerous growths, and upon the appearance of new diseases, such as osteomegalacia (indicating an abnormal rapidity of cell-growth), among people who practice vaccination.

Q. 4,274. "The lymph is partly used for calf to arm vaccination at the station, partly sent up to the Local Government Board."

Q. 4,278. "The origin of the lymph was some sent over to England by Dr. Dubreuille, who obtained it from a village called Laforet, near Bordeaux."

Q. 4,279. (The Chairman.) "Do you know whether that was from the natural cow-pox?" "That was from what is generally termed spontaneous cow-pox; that is as it occurs first in the cow."

Q. 4,280. (Prof. Michael Foster.) "And has passed through the cow only?" "That is so. The lymph that we are using has never been humanized."

(See *post*, Qs. 4,586, 4,587, 4,600, 8,900, 8,923–29.)

Q. 4281. (The Chairman.) "Has that lymph been used during the whole time?" "No; before March, 1882, the lymph stock was established by Dr. Carsten, of the Hague, and at that date the stock was exchanged for the Laforet lymph." * * * "For a limited time, another stock of lymph was set up alongside the other, for the sake of comparison."

Qs. 4282, 4283. "It was supplied by Dr. Simpson, of Aberdeen. I think it came from the calf."

Q. 4,284. "We used this lymph on the guarantee of Dr. Simpson, who was afterwards medical officer of Calcutta. Dr. Cory is not able to speak definitely as to its origin. The guarantee was that it was calf lymph, and had not passed through any human being."

Qs. 4,288, 4,289. This lymph has been given up, because "the vesicles produced by the lymph that we obtained from Dr. Dubreuille were better than those produced by Dr. Simpson's lymph."

Dr. Cory then described his duties as instructor in vaccination.

Q. 4,300. (By the Chairman). "We have been told that besides the lymph distributed in tubes of the Local Government Board, some lymph is distributed on points which are received from you; how is that lymph obtained?" "That lymph is obtained from children at the station where we use human lymph."

Q. 4,202. "Will you tell the Commission how that lymph is put upon the points?" "The child's arm is pricked, and then, unless blood flows (we are always very careful not to take any blood upon the points), the points are dipped into the lymph that wells up in little globules, and the points are allowed to dry for ten or twenty minutes."

Q. 4,303. "In the ordinary air."

Q. 4,311. (By Sir James Paget.) "You spoke of the lymph being more or less good; is there any test of the goodness of the lymph except the kind of vesicle and scar which it produces?" "Of course I am only giving now my own individual opinion. *I believe that is the only test you can get.* There are also the microscopical tests which are used, and their tendency is, no doubt to cause the rejection of a great deal of good lymph." (*Post*, Q. 4,649.)

Q. 4,312. "Has it ever been observed that small-pox has prevailed in a district where one kind of lymph has been used, and not in another, where another kind of lymph has been used?" "Not that I know of."

Q. 4,313. "You cannot tell the difference between a child vaccinated with lymph from the calf and that from a child."

Q. 4,314. "Is the calf lymph that you have now at Lamb's Conduit Street, entirely derived from that one source that you speak of in France?" "Yes."

Q. 4,315. "With the exception he spoke of, they have never had it from any other natural cow-pox" (Q. 4,316) "except a source got last year from Gloucestershire, carried on for a short while side by side with the ordinary stock, and used in the vaccination of one or two children."

Q. 4,317. "Nor have you ever had it, I suppose, from a cow that had been variolated?" "No; some attempts of my own at direct variolation of the cow and calf were made elsewhere than at the station."

Q. 4,318. "Have you used lymph from a cow that had been inoculated with small-pox?" "I believe that was the origin of Simpson's lymph."

Q. 4,320. "From a cow which had been inoculated with small-pox?" "Yes." Q. 4,321. "And then carried through several children?" "No; at first through the calf."

Q. 4,322. "From the cow to the calf, and then by successive removes from calf to calf?" "Yes; I should say that the lymph we afterwards used upon children *never produced anything but vaccine.* There must have been a great many children vaccinated from the lymph, and it never produced any other effect but vaccine."

Q. 4,323. "You mean by that the eruption?" "Yes; the places on the arm, and they were not small-pox. And there is no instance of contagion having taken place from one child to another at that time after the manner in which small-pox is propagated."

Qs. 4,324, 4,325. "Assuming this was a case derived from a cow which was inoculated with small-pox, and the lymph then transferred to the calf, and then from one calf to another, nothing was produced in children but the ordinary vaccinia. There never was any suspicion of other pustules about."

Qs. 4,327, 4,328. "He knew only by reading of any other case of the variolation of the cow. He had tried it himself, but has never succeeded in producing any vesicle on the bovine animal." (Q. 4,329.) "But he believes that Dr. Simpson's lymph was derived from a cow which had been inoculated with small-pox," and (Q. 4,330) "he never saw any mischief following."

Qs. 4,331–4. (By Mr. Savory.) "The statement that Dr. Simpson's lymph was due to the small-pox inoculation of the cow was made from notes takon by Dr. Murphy at the time of the information then obtained from Dr. Simpson, and witness knows of no other case of a similar experiment being performed successfully, except from general literature."

On the 4th of December Dr. Cory appeared before the Commission, and began by explaining much of his previous testimony; and with regard to this lymph obtained from Dr. Simpson, he reads the notes of his assistant, Dr. Murphy:

Q. 4,558. "On 21st of November, 1885, inoculated a calf with lymph on five points, supplied by Dr. Simpson, of Aberdeen. Lymph used had been taken from a vesicle which formed on a cow's teat after inoculation with small-pox lymph from an unvaccinated patient."

"On 26th of November three of the five places of insertion have some *appearance* of vesiculation."

"Calves and children inoculated from these appearances developed normal and well-formed pearly vesicles."

Q. 4,564. (Dr. Collins.) "In the notes of Dr. Murphy, which you have read, there is no statement as to the actual experiment of inoculating the cow with the small-pox; could you tell us who performed that experiment, and where?" "It was performed by Dr. Simpson, in Aberdeen." Q. 4,565. "By himself."

Q. 4,566. "Is that stated in these notes?" "*No; it is not.*"

Q. 4,567. "That has been separately ascertained?" "*I believe so.*" [*Credo qüia impossibile.*—Ed.]

Q. 4,568. "Has any publication of the experiments which led to the raising of this stock of lymph been made?" "No; I do not believe it has been published at all."

Here, then, we have the Dubreuille lymph, said to be from a case of "*spontaneous*" cow-pox (declared by Jenner to be spurious), and a lymph *said to be* produced by inoculating a cow with small-pox; both used indiscriminately as "calf lymph," both said to produce vaccine (Q. 4,322), and, as will be seen presently, the Dubreuille lymph, from which the British calf lymph is chiefly derived, has been discontinued in France, because of doubts as to its genuineness; of all which, however, Dr. Cory had never heard, nor made any inquiry concerning it. (Q. 4,586 and subs.)

To return to the proceedings of the 27th of November, Dr. Cory, still under examination of Mr. Savory:

Q. 4,335–6. "Whether a child was vaccinated with lymph obtained directly from the calf, or with lymph obtained from another child, witness would be unable to distinguish the difference." "The result is practically the same."

Qs. 4,337, 4,338. "Dr. Cory has vaccinated a little under 50,000 children, and, in his opinion, it was a matter of comparative indifference which lymph was used."

Qs. 4,339, 4,340. "He has no choice, and thinks it a matter of absolute indifference" (see *post*, Qs. 4,590, 4,591, 4,592, 4,598, and 4,601.)

Q. 4,342. "Has often heard that when a child is vaccinated directly from the calf, the results are more severe than when it is vaccinated from another child, and thinks that the reason is, that the calf lymph generally used in England has been got from Dr. Warlomont,* and that he takes his lymph on the eighth day. The lymph, when you take it late from the calf, produces inflamed vesicles."

Q. 4,344. "Considers the best time for taking lymph for vaccinating to be on the eighth day from a child, and on the fifth day from a calf."

Q. 4,345. "Supposing that the operation is delayed (as it very often is perhaps to the ninth day), do you think that the lymph deteriorates materially in the 24 hours?" "I am desirous of protecting lymph from organisms that may get access to it. I am now speaking of calf lymph. When a cow is vaccinated there is a lot of straw about, and from that foreign organisms may get upon the lymph and give rise to abnormal results. So I would not risk the rupture of a vesicle."

Q. 4346. "So far as the lymph is concerned that would not make a material difference?" "No."

Q. 4.673. Quotes Q. and A. 4,345.

Q. 4,674. "What results do you apprehend are referable to the organisms which are derived from the strain?" "When the vesicle is rubbed and the organisms get entrance into it, you might get suppuration of the part."

"Q. 4,675. "Are we to understand you as offering that explanation of the inflammation of the vesicles occurring in the use of lymph taken later than the fifth day?" "When the vesicles are older than the fifth day they have a greater tendency to be broken by the animal lying down, and when the head of the vesicle is rubbed off, the organizations have opportunity of entrance; but the vesicle is always washed with a disinfectant

---

* Warlomont lymph is *claimed* as the origin of the New York lymph.

lotion before we take the lymph, *organisms are not in the vesicles then*."

Compare this "opinion" with the *facts*, as *observed* by Dr. Crookshank and described in his evidence. He found that *none* of the "pure lymph" of the Local Government Board was free from *pathogenic bacteria*.

The same was found to be true of American (pure) lymph, by Dr. Walter Reed, U. S. A., a believer in the virtues of vaccination.

Q. 4,347–9. The reasons for taking the lymph from the calf on the fifth day and from the human subject on the eighth, are because the changes are more rapid in the vesicle in the calf than in man, and that the temperature of the calf is one degree and a fraction higher than that of a human infant, which hastens the changes in the vesicle. This is not a matter of inference, but of observation.

Q. 4,350. (Prof. Michael Foster.) "The whole thing goes on more rapidly in the calf?" "It does."

Q. 4,351. "There are other differences to be considered in the calf besides temperature which may also have to do with this."

Q. 4,353–6. "The L. G. B. lymph is a different strain from Warlomont's. Warlomont's came from Italy; it was brought by Lanois from Naples."

Q. 4,357. "Do you know that your strain will, as you say Warlomont's does, produce more inflammation if the lymph is taken on the eighth day?" "I do not insist upon that. I have used Warlomont's lymph and I know that that does produce inflamed arms."

Note here and in numerous other parts of the testimony of Dr. Cory and of the other vaccinal witnesses the callousness to the suffering inflicted upon little children—*always mark the children of the poor*, which the above remark illustrates. Warlomont's lymph was used—the *experiment* was tried—upon whom? and *inflamed* arms were produced. That is, painful arms.

And yet in Great Britain, as in New York and other States of the Union, societies exist which arrogate to themselves the name of societies for the *prevention* of cruelty to children! Is there any cruelty which has come to their notice equal to that of causing "inflamed arms" to millions of little children—producing ulcers upon them by inoculating them *with the putrefying matter of a sore*, often wrecking their lives, whereof one of the many thousands of instances is here produced? And yet not one of these societies with pretentious names and aristocratic managers has ever made the slightest effort to stop these cruelties to children!

This is a portrait of John Pfænder, a child of healthy Swiss parents, born 23d September, 1875, was healthy until vaccinated. Was a sturdy and beautiful child, as were also his four brothers and sisters; he walked at the age of nine months.

On the 16th June, 1876, he was vaccinated by the official vaccinator. Eight days later his feet began to swell, abscesses formed, his teeth began to rot, his glands to swell. Fistulous sores appeared on his hands and feet. The above photograph was taken in May, 1882. He could neither walk nor stand; several of the bones of his hands had rotted out.

It was such cases as this and the spirit of liberty, not yet dead in Switzerland, which led that freedom-loving people to reject on a referendum, the infamous system of blood-poisoning, ycleped vaccination, which a medical clique was seeking to impose upon them.

When will the good sense, parental affection, and love of liberty of Americans rise to the level of that of the brave burghers of Switzerland?

There are thousands of such cases in this country.

Q. 4,360. "In your case you do not find that amongst the infants vaccinated with calf lymph there is greater inflammation, or greater tendency toward untoward results, such as ulceration, than with humanized lymph?" "I do not believe you would, at *the date of inspection*, tell the difference between the effects of the humanized lymph and the calf lymph," and (4361) "that has been the case from the beginning." (See *post*, 4491.)

Q. 4,363. "Of the witness' own vaccinations over 15,000 have been performed with humanized lymph, and about 32,000 with calf lymph."

Qs. 4,364–5. The calf lymph has been going on since 1881, and it was begun in 1882 with the seventeenth remove from the (so-called) spontaneous case.

Qs. 4,367–8. A description of that case was given by Dr. Herrineux, in the *Weekly Gazette of Medicine and Surgery*, of Paris, on the 20th of January, 1882. (See *post*, 4,578–9.)

Qs. 4,369–70. His experience leads him to reject the view that lymph taken from the cow leads to greater inflammation and has a greater tendency to produce ulceration than lymph which has been humanized. (See *post*, 4,491.)

Witness presents the number of cases of calf-lymph vaccination at the calf-lymph station from 1882 to the end of September, 1889, being altogether 32,000.

Each parent receives two cards, specimens whereof were produced. The red card is given on the day the vaccination is performed, and the black is given on the day week. As to the red card see *post*, 4,509–11. On the black card is this direction:

"If, as sometimes happens, owing to the child's state of health the arm does not heal up readily, the child shall be brought back to the station."

Q. 4,372. "No cases were brought back till 1884 to 1885."

Q. 4,373. The cases then brought back were calf-lymph cases. "Between 1884 and 1885 there were 2,901 cases, and 22 cases were brought back. From 1885 to 1886, out of 4,054

cases, there were 37 brought back with complaints. From 1886 to 1887 there were 5,591 cases, and 64 were brought back. Between 1887 and 1888, out of 6,151 cases, 80 were brought back. From 1888 to 1889 there were 7,067 cases, and 60 were brought back. From April, 1889, to the end of September, there were 4,330 cases; 60 were brought back with complaints. So, out of 32,000, the number brought back for complaints was 1.009 per cent."

Qs. 4,374–5. "We have no similar table for human lymph."

Q. 4,376. "Were any of these cases serious ones?" "I classify them thus: Sore arms, eruptions, erysipelas, and axillary abscess. Out of the 323 cases brought back, 260 of them were for sore arms, unhealed arms."

Q. 4,380. *There was some induration* in that class of cases.*

Q. 4,381. (Chairman) "Could any of these cases have arisen, from the way they had been treated, from rubbing off or want of care?" "The majority of them are occasioned by the mothers putting on applications which they had better not put on. There is 'Lion's plaster,' for instance. I do *not know what* it consists of; that is a favorite method of treating vaccine vesicles. You would be astonished to see the hideous stuff some people will make up in a pot and put on to the places; *they cannot let the vesicles alone*."

That is to say a mother cannot see her child suffer without making some effort to relieve its pain. Is any stuff they put *on more* "hideous" than the *putrefying matter of a sore* which the doctor has put *in?*

Q. 4,382. (Professor Michael Foster.) "To the best of your belief, if those arms had been properly treated they would have healed up in due time?" "Yes, in the great majority of such cases they would have healed up in due time if they had been left alone."

How does he know that? He has not even taken the pains to find out what the poor mothers' "favorite method of treating vaccine vesicles, Lyon's plaster," consists of!

* It is well to bear in mind *that this* is one of the characteristics of the syphilitic chancre. Never of small-pox.

Q. 4,383. (Sir James Paget.) "You ascribe the sore arms to the treatment, rather than to the vaccination?" "Yes."

Then 38 are returned for eruptions of various kinds. (See *post*, Q. 4,704.

What kind of scientific intellect is this which attributes a result to a "treatment" of which he knows nothing, instead of seeking the cause in what he knows to be the *putrefying matter of a sore poured into the blood*, and which in the hands and under the watchful eyes of the highest vaccinal authorities has produced the very results he now ascribes to this "treatment" whereof he avows he knows nothing.

Q. 4,384. (Prof. Michael Foster.) "Is there anything special about those eruptions?" "No; eczema and lichen."

That is, diseases occasioned by a diseased condition of the blood, they are common secondaries to syphilis.

[See *Wilson on Skin Diseases*, Chap. XVII, Syphilitic Eruptions; pp. 801–803.]

Q. 4,385. "There is one eruption Dr. Cory thinks peculiar to vaccination—something between an urticaria and an eczema. Little blisters are formed on the surface of the skin. Has seen children have it very severely.

Q. 4,387. The child may have a return of the eruption during dentition.

Q. 4,390. "Out of the 32,000 cases there were 260 with sore arms, and 38 with eruptions. Then there were 16 cases of erysipelas and 9 of axillary abscess." *

Q. 4,392. (Mr. Savory.) "These were attacks; were there any deaths?" "Eight deaths were reported to the station of children who had been vaccinated. The first one was vaccinated March 3d, 1885, and the date of death was March 9th, 1895, the death being certified as convulsions. In the case of the second death the child was vaccinated on the 28th of April, 1885, and died on the 12th of May, from confluent small-pox; it was vaccinated late in the incubation period of the small-pox; the small-pox appeared about the fifth day after vaccination."

---

* It should be remembered that according to Jenner erysipelas was essential to a successful vaccination. If there were no erysipelas the cow-pox was "spurious."

Q. 4,393. "That could not be ascribed to vaccination?"* "No. These are the eight cases of death reported to the station, and vaccination was accused of the death in each of the cases."

With reference to Q. 4,390 see *post*, Q. 4,686.

Qs. 4,395–6. "As to the next death, the child was vaccinated on the 13th of August, 1885, and died on the 10th of October. I heard from Dr. Webb that it had died, and that death was attributed to vaccination, but I did not ascertain what it had really died of."

Q. 4,397. (Prof. Michael Foster.) "The cause of death is not stated. No. Then in the next case the child was vaccinated on the 2d of February, 1886, and died on the 8th of February, 1886. That was from inflammation of the bowels. Of course the mother very often, when a child dies, blames vaccination for doing that which vaccination has no business to be blamed for.† * * * Then in the next death the child was vaccinated on the 23d of November, 1886, and died on the 7th of January, 1887, from cellulitis *that may have been from vaccination.*"

Q. 4,398. (Sir James Paget.) "Do you know anything of what occurred intermediately between vaccination and the occur-

---

* Why not? Wilson says (*ubi supra*, pp. 96, 97): In very severe cases the period of incubation is short; in the milder forms, on the contrary, it is long. The limits commonly assigned to this period are from 5 to 20 days. There can be no question as to the severity of this case, for the child died, and Dr. Cory says the small-pox appeared about the fifth day after vaccination; that would give vaccination as the starting point of the disease; five days for the period of incubation, and nine days for invasion, eruption, suppuration and DEATH. Nor is the statement that the child "was vaccinated *late* in the incubation period," deserving of respect, for in such case the child would have exhibited symptoms of ill-health at the time Dr. Cory vaccinated him, which, under the rules of the Local Government Board, to which Dr. Cory, above all persons, must be supposed to have given close obedience, would have prevented its being operated upon at all.

† And the mother knows best. Her child is healthy; it is made sick by having inserted into its blood the *putrefying matter of a sore;* it continues sick; its vitality is lowered, and among the infinite variety of forms which the artificially-produced sickness may take, inflammation of the bowels is one. The child dies, and the mother *knows* that vaccination killed it, in spite of all the euphemistic rigmarole with which the vaccinists may seek "to preserve vaccination from reproach." (See letter of Dr. May in the Birmingham (England) *Medical Review*, January, 1874.)

rence of the cellulitis?" "No. I made inquiries. I could not acquit vaccination entirely of the death in that case. Then in the next death the child was vaccinated on the 5th of April, 1887, and died on the 12th of April, 1887, from *convulsions*. Then in the next death the child was vaccinated on the 8th of October, 1889, and died on the 1st of November, 1889. That was from erysipelas, and in the last death the child was vaccinated on the 22d of October, 1889, and died on the 20th of November, 1889. That was from convulsions. I investigated these two last cases myself. I did not see the child that died of erysipelas. The erysipelas began in the second week, and seems to have proceeded from the vaccination wound. The other case *was merely* a case of convulsions, which *might* have occurred from bowel complaint, not from vaccination at all."

Dr. Cory seems to regard the human organism as made up of separate parts, any one of which may get out of order and be repaired without affecting the rest of the machine.

Q. 4,406. (Prof. Michael Foster.) "Do the facts show that your calf lymph, which has never been humanized and has descended, to the best of your knowledge, from a case of spontaneous cow-pox, does not, supposing the lymph to be taken on the fifth day, give rise to any unusual inflammation or any unusual bad results?" "Yes, as compared with standard arm-to-arm lymph, this is what the facts do show." (See *post*, Q. 4,491.)

Q. 4,417. "There is a difficulty, is there not, in extracting the lymph from the vesicle of a calf, as compared with a human vesicle?" "Yes, we have to use clamps." Is the Society for the Prevention of Cruelty to Animals asleep, or are its directors hypnotized by the grandeur of the official calf-torturers?

Q. 4,423. "Do you get clear lymph in that way, free from blood?" "No; it is always mixed with blood, more or less."

Qs. 4,435–9. Dr. Cory has attempted bovine inoculation five times without success. He got papules similar (he believes) to those got by Dr. Chauveau.

Qs. 4,446–9. He does not believe that any children under ten years of age are insusceptible to vaccination. He has always succeeded in vaccinating children reputed insusceptible.

Q. 4,451. (By the Chairman.) "There were 22 cases in 1888 out of 5,005, in which he failed the first time, and afterwards succeeded, and that is about the proportion in all."

Q. 4,455. (By D. Hutchinson.) "Dr. Benner is the owner of a private calf-lymph establishment. He takes his calf-lymph on the fifth day."

Qs. 4,457–9. "He (Dr. Cory) has met with some cases in which the sores took three weeks to heal."

Qs. 4,460, 4,461. "He would expect to find enlargement of the axillary glands—a common consequence of vaccination. He has seen axillary abscesses in nine cases." *

Qs. 4,462, 4,463. "The eruption occurring sometimes after vaccination does not always keep to the type described (*supra*, Q. 4,385). There are considerable varieties in the eruptions following on vaccination."

Qs. 4,465, 4,466. "He does not remember Dr. Gregory's description of it. It is usually vesicular, sometimes on the backs of the hands and feet."

Q. 4,467. "When what you call a lichen eruption comes out without a vesicle, do you consider that vaccinia eruption?" "It is provoked by vaccination. Or, to take another instance, you may get a syphilitic eruption in a child after vaccination, seen on the tenth day. You never ascribe a secondary rash of syphilis appearing after only ten days to an inoculation. * * * Vaccination being a cutaneous irritant, it has produced an eruption in an already diseased child; that, no doubt, is the reason of syphilis appearing so early after vaccinating, just in the same way as a person playing foot-ball gets a kick, and the (syphilitic) node. I look upon a perfectly normal skin as not being affected by vaccination; it is only the tender skin that gets a lichenous or other rash after vaccination."

Q. 4,662. (Dr. Collins.) "* * * Would that explanation apply to all syphilitic eruptions that followed vaccination?" "Unless the syphilis was conveyed by the vaccination; but then

---

* In syphilis we have buboes: That is, abscess in the packets of glans nearest to the place of infection.

you would not get the syphilitic eruption appearing on the tenth day after the vaccination."

Q. 4,663. "So that that explanation only applies to those recent cases in which the eruption follows within a week or two after the vaccination?" "Quite so, to cases of inherited syphilis."

Q. 4,468. (Mr. Hutchinson.) "A true vaccinia eruption has nothing to do with the health of the child?" "*No; not as injuring the child!*"

Q. 4,469. "What precautions do you recommend to be taken as regards the protection of vaccination sores?" "We would rather not do anything to them."

Q. 4,470. "Are no instructions given to the parents of the child as to what should or should not be done?" "We tell them not to use shields, and recommend them to put upon the sores a little clean piece of linen rag."

Q. 4,476. (Still by Mr. Hutchinson.) "I take it that there were only eight deaths that you know of in the 32,000?" "Yes."

Q. 4,482. Dr. Cory does not think that a syphilitic taint has been the cause of sore arms or of abscesses in the axilla.*

Qs. 4,483–8. (By Mr. Whitbread.) The rule as to rejecting lymph because of the presence in it of blood does not apply to calf lymph, Owing to the method of obtaining it by means of clamps, which are employed of necessity, most of the calf lymph contains blood.

Qs. 4,489–90. He believes that the number of sore arms is greater in vaccination from calf lymph than from humanized lymph; but he has not ascertained the number brought back with sore arms after the use of human lymph.

Q. 4,491. Dr. Cory repeats his impression (in answer to Mr.

---

*As the annual vaccinations in England and Wales range from about 400,000 to about 700,000, take 550,000 for an average, and we should have innocent victims to the number of 136 yearly put to death by vaccination. Suppose, now, 136 persons were to be yearly offered up as sacrifices to propitiate some deity and to secure its protection from some one disease, what would we think of the enlightenment of the people perpetrating such acts? In reality, the number thus offered upon the altar of the God cow-pox is far greater than are borne upon any official returns, while the number whose lives are wrecked by inoculated disease, or who have their vitality lowered by the sepsis of vaccination, is incalculably greater.

Meadows White) that you get more sore arms after using calf lymph, but states there has not been the same care in registering the cases.

Qs. 4,492–3. (By Mr. Whitbread.) Is of opinion that organisms get into calf lymph after the vesicles are broken, and regards the eighth day as too late to take lymph from the calf. He has never subjected lymph taken on the eighth day to microscopical examination.

Q. 4,494. His opinion is based upon the fact that there are a lot of organisms in hay and straw, and as time goes on, those elements which it is desired to avoid have increased opportunity of access to the lymph.

Q. 4,495. There is the fact, as a condition of calf lymph that the calf is of a higher temperature.

Qs. 4,495–6. "If there was a considerable difference in calf lymph on the eighth day as compared with the fifth, would it probably show itself under the microscope?" "I do not know."

Q. 4,498. (To the Chairman.) It is a matter of observation that the vesicle has progressed further on the eighth day in the case of the calf than in the case of the human subject.

Q. 4,501. (To Prof. Michael Foster.) The calf on the eighth day has about reached the same stage as that reached on the twelfth day in the child.

Q. 4,502. (To Sir James Paget.) The more irritating effects of vaccination are produced by later takings of the lymph, and (Q. 4,503) the more quickly the cycle which reaches to a perfect vaccine vesicle is finished the more mild is the lymph produced. The calf lymph taken at the fifth day is as effective and less irritating than calf lymph taken on the eighth day.

Q. 4,509. (Mr. Whitbread.) "I see this statement at the foot of the red card:

"Nurses of small-pox hospitals are always re-vaccinated before commencing their work. This has been the rule for over fifty years. None of them have taken small-pox since this rule has been observed." Does that mean that no nurse at a small-pox hospital has taken small-pox for the last fifty years? That card gives the Highgate Hospital experience and was issued without reference to the Asylums Board Hospitals. I do not know how the final 's' came to be inserted."

Q. 4,511. "Nurses of small-pox hospitals are always vaccinated before commencing their work. This has been the rule for over fifty years. None of them have taken small-pox since this rule has been observed." That would convey to my mind that no nurse for the last fifty years in one of the small-pox hospitals has taken small-pox. "If they have been re-vaccinated and the re-vaccination has been completed before a nurse goes on duty, I believe your impression expresses the fact."*

Q. 4,554. (Chairman) "We understand that you desire to make some explanation or correction of your former evidence?" "Perhaps I may be allowed to say one or two things in the way of correction with regard to the evidence that I gave last Wednesday. I should like first of all to hand in these four cards. I may explain how the word 'hospitals' was introduced into the card instead of 'hospital.' It was originally printed 'hospital,' but through my oversight in looking over the proof of a reprint of the card I quite inadvertently let the 's' stand.

"This is the last printed card and the only one of the whole series that contains the plural 'hospitals.'"

Q. 4,555. "In the earlier card the reference was exclusively to what is known as the Small-Pox Hospital at Highgate, which is the only small-pox hospital that has existed for any great length of time?" "Yes, quite so."

Q. 4,556. The statement in the card would be true of the Small-pox Hospital at Highgate, and of all the London small-pox hospitals, *if you deduct all the cases that were vaccinated too late.*

---

*"Quackery has always one shuffle left," was the remark made by William Cobbett with regard to Edward Jenner and his repeated excuses for failure after failure to "protect." It will be as well to give here the further "explanation" of Dr. Cory; given on the 4th December, 1889.

## Figures and Tables

**Figure f1:**